# Urban Greenspace, Climate Adaptation and Health Co-Benefits: Municipal Policy and Practice in London

**DOI:** 10.3390/ijerph22030409

**Published:** 2025-03-11

**Authors:** Catalina Turcu

**Affiliations:** Bartlett School of Planning, University College London, 14 Upper Woburn Place, Central House, London WC1H 0NN, UK; catalina.turcu@ucl.ac.uk

**Keywords:** urban greenspace, climate adaptation, health co-benefits, local government, policy integration, London

## Abstract

Climate change poses a significant threat to human health and wellbeing, yet its health impacts can be mitigated through effective local action. Green spaces offer numerous climate benefits to cities, including improving air quality, water management and providing local cooling effects, with subsequent health benefits. Despite such benefits, the current municipal policy and practice faces challenges in aligning climate, health and greenspace interventions on the ground. This paper looks at the municipal evidence base in London. Employing a policy-engaged approach, it draws on semi-structured interviews and focus group discussions with London boroughs to unpack what greenspace indicators are measured and why; what feeds into municipal evaluation frameworks; and how greenspace, climate adaptation and health are integrated across London’s municipalities. The findings reveal limited and fragmented approaches to measuring the multiple benefits of greenspace interventions, with weak links to climate and health outcomes, and little policy alignment at the municipal level. This has broader implications for data-driven governance models pursued by cities worldwide and for integrating greenspace–climate–health policy and practice within the spatial and political context of cities. The paper concludes by summarising research findings, presenting policy recommendations and highlighting areas of future research.

## 1. Introduction

Climate change presents a fundamental threat to human health and wellbeing, especially in cities where populations are densely concentrated. Many of the health impacts of climate change in cities, such as heat- or air pollution-related illness but also mental health and wellbeing conditions, are avoidable with appropriate climate measures. A city’s greenspace or green infrastructure—broadly defined as a system of natural and artificial greenspaces that provide ecological and social functions in urban areas and ‘greenspace’ thereafter—has multiple benefits [1]. The health benefits of greenspaces have been extensively reported in the literature; these include improving overall physical health by encouraging physical activity and mental health and wellbeing outcomes by providing relaxation, stress and anxiety alleviation and social interaction [2]. Greenspace also provides climate benefits such as reducing air pollution by using and storing carbon and capturing other greenhouse gases, managing flooding by absorbing excess surface water and lowering urban heat by providing shade and cooling [3].

The multifunctionality of greenspace frames academic debates on ecosystem services, nature-based solutions, biodiversity, nature-positive economy and biophilic design, among others, and greenspace is viewed as part of the ‘solution’ to global crises such as climate change and the COVID-19 pandemic by contributing to more sustainable, resilient and healthier places to live [4]. This is paralleled by significant technological innovation, such as remote and smart sensors mapping the natural environment and data-driven tools to model, plan and monitor greenspace development, while strong intergovernmental and national policy agendas, such as UNEP’s ‘making peace with nature’ [5] and UK’s ‘restoring nature’ and ‘biodiversity net gain’ strategies, incentivise cities and local governments around the world to embrace policies and interventions that promote urban greenspace [6].

Hence, urban scholars and policymakers are increasingly advocating for greenspace as part of nature-based solutions’ mantra to enhance adaptation responses to climate change [7]. A recent systematic review found that 90% of studies focus on greenspace as an adaptation measure, especially in flood management and heat reduction [8]. There is also recognition that aligning any type of urban intervention, including greenspace intervention and climate adaptation measures, needs policy integration in practice [9], with a newly emerging body of literature content that such an alignment also results in substantial health co-benefits [10,11]. However, there is little understanding of how greenspace adaptation is implemented in practice and how it aligns with wider public health goals, with many cities acknowledging gaps between policy and practice due to a lack of resources, political leadership, land availability and siloed governance structures [6]. This paper seeks to further develop such understanding and asks:

*What is the current practice of urban greenspace adaptation with health co-benefits at the municipal level?*

More specifically, it aims to explore the current evidence base in London to unpack (1) what greenspace outcomes are measured; (2) what feeds into municipal evaluation frameworks and (3) how greenspace, climate adaptation and health policy and interventions are integrated across London boroughs. London is a city of global relevance. Its climate vulnerabilities, including air pollution, flooding and heat, impact on population health and determine health inequalities across the city [12]. Hence, increasing and enhancing London’s greenspace is viewed as both an important climate adaptation measure and public health asset, for example, it has been estimated that London’s trees helped avoid 153 heat-related deaths in 2015–2022, and an increase in London’s tree canopy cover by 10% would reduce heat-related mortality by 10% [13].

The paper makes three significant contributions to the field. First, it adds to an emerging body of literature on climate action with health co-benefits in cities [10,11], with a specific focus on the multiple benefits of greenspace; it also advances prior studies that called for a better understanding of the urban and spatial aspects that inform climate change and public health interventions in practice [3]. Second, it provides valuable insights into contemporary municipal understanding and practice. The persistent ‘great gap’ between research and practice has been well documented since the early 2000s, and this is also the case for urban greenspace interventions from the perspective of the climate–health nexus [14]. Third, while environmental and public health research have traditionally emphasised quantitative and modelling approaches, this paper adopts a complementary perspective by utilising qualitative and policy-orientated methods. This approach fosters a more nuanced and contextually grounded understanding of environmental and public health challenges, ultimately contributing to the development of more equitable and actionable solutions.

### 1.1. The Multifunctionality Thesis

There is no agreed-on definition of greenspace in the literature; however, two broad interpretations are used: greenspace as synonymous with nature and greenspace as explicitly urban vegetation [15], with the latter being used at the municipal level. It refers to any natural and semi-natural features such as parks, urban forests, green roofs, rain gardens, permeable pavements, wetlands and vegetated corridors that are in the city. Urban greenspace provides multiple environmental, economic, social and health benefits [16,17]. It provides important contributions to climate adaptation, biodiversity and ecosystem services, and it is even more important in cities due to land use competition [18]. It can contribute to the circular economy by providing bioproducts (e.g., food, compost, timber, bioplastic, etc.) and create job opportunities. Urban greenspace is important for quality of life and socialising [19]. It can also encourage physical activity, with positive effects for physical health outcomes, especially in the elderly, children, pregnant women and disabled people, but also improve mental health and wellbeing outcomes by reducing stress and anxiety and supporting better cognitive development in children [3].

Existing studies have suggested that greenspace interventions can achieve ’multifunctionality’, whereby multiple benefits are produced concurrently. This has political appeal and plays on a broad scope of influence to enhance climate adaptation action. The multifunctionality thesis positions greenspace as a ‘no regrets’ option and a strategic platform for integrating climate benefits with co-benefits for broader sustainable development, including health co-benefits [8]. It has informed urban solutions such as sustainable drainage systems (SuDS) and rooftop gardens, for example, to innovate beyond traditional greenspace approaches to manage urban flooding and associated health risks and to support urban agriculture while mitigating the overheating effects. However, barriers to greenspace’s multifunctionality exist. They stem from complex urban governance dynamics but also stakeholders’ limited awareness of its multiple benefits [8] and can led to ‘maladaptation’, whereby actions intended to reduce climate vulnerability cause adverse impacts or increase vulnerability in/across other systems, sectors or social groups [20]. Examples include the removal of trees in cities to reduce the risk of wildfires, which, in turn, can impact negatively on health (e.g., by reducing air quality and increasing exposure to urban heat island effects), increase the risk of flooding and contribute to biodiversity loss. In current practice, urban greenspace is used mono-dimensionally rather than being recognised for its multiple benefits, and its significant synergies with climate change and public health are often overlooked [1].

### 1.2. The Greenspace–Climate–Health Nexus

As a climate adaptation strategy, greenspace provides numerous benefits that enhance a city’s capacity to anticipate and manage climate risks while simultaneously addressing a variety of health exposures linked to climate change. Greenspace adaptation delivers significant health co-benefits, such as improving outcomes and preventing the onset of new heat and air pollution-related conditions but also healthcare savings by reducing hospital admissions [21]. Sharifi et al. (2021) noted that greenspace and nature-based solutions associated with climate adaptation yield some of the highest health co-benefits [22]. At the same time, greenspace contributes to health promotion in the city through its positive associations with physical activity and better mental health and wellbeing [3].

However, the current research addresses the environmental or health impacts/benefits of greenspace separately, and there is limited understanding of, for example, how greenspace adaptation may influence health outcomes [23]. The existing studies can be broadly categorised in two groups: studies examining the health co-benefits of climate adaptation measures, with greenspace interventions as an example, and studies focusing on the multiple benefits and co-benefits (including health) of greenspace-related climate adaptation [8,22]. The former family of studies often provides insufficient detail on greenspace-specific interventions, while the latter one fails to adequately emphasise the health co-benefits of greenspace, derived from climate-related action. Moreover, we know little about how health co-benefits of greenspace adaptation are gauged, quantified and monitored, particularly in relation to mental health and wellbeing but also managing chronic conditions like diabetes, cardiovascular and respiratory disease and cancer [3,24].

### 1.3. The Policy–Practice Gap

A gap already exists between urban adaptation plans and their implementation [25]. While several cities have successfully implemented local greenspace adaptation plans, these initiatives often fail to explicitly integrate public health considerations [9]. Moreover, despite the well-documented benefits of utilising greenspace for climate adaptation, its adoption has been relatively slow [26]. There are two possible explanations for this. First, greenspace is often perceived as ‘nice to have’ rather than a necessity because of its long-term maintenance costs. This view undermines recognition of its critical environmental, ecological and social functions [27,28]. Despite policy emphasis on greenspace, funding for such interventions has faced significant challenges in recent years due to either austerity measures or declining public budgets. Financial constraints have spurred efforts to identify alternative funding mechanisms and have ignited debates about the roles and responsibilities of various stakeholders, including local governments, private developers and community groups. Mell (2020) highlighted several alternative funding approaches, such as developer contributions, business improvement district levies, corporate social responsibility (CSR) initiatives and community-led funding models [29]. Moreover, sociocultural and political–institutional considerations such as public acceptance, social dynamics, cultural views and governance structures can pose significant challenges to the delivery of greenspace interventions, and different urban stakeholders engage with greenspace delivery according to their agendas and interests, further complicating efforts to achieve cohesive and effective outcomes [30].

Second, implementing greenspace adaptation with health co-benefits involves collaboration between many different policy sectors, including public health, climate, spatial planning, conservation and so on, which need to come together and integrate their efforts to achieve greater action as a group [24]. This is no small task. Urban governance structures are characterised by cumbersome bureaucracies and path-dependant decision-making processes that can challenge effective climate and health risk management in urban settings [31]. Moreover, current policy discourse is often fragmented [32] or issue-driven, with various stakeholders holding distinct responsibilities, which can lead to a lack of coordination and integration [33,34]. Sharifi et al. (2021) noted the necessity of robust governance and institutions to harness the health co-benefits of greenspace adaptation, emphasising the inclusion of the health sector in climate change and greenspace decision-making processes [22]. This implies overcoming institutional barriers and requires coordinated efforts to integrate policies across different sectors [35], promote knowledge exchange among diverse stakeholders and envision transformative scenarios for a more holistic approach to improving the city [36].

The paper is organised into four further sections following from this introduction. Section 2 outlines the materials and methods, followed by the presentation of the results in Section 3. Section 4 develops the discussion of the results, and Section 5 concludes the paper by summarising the main findings, making policy recommendations and highlighting areas of future research.

## 2. Materials and Methods

### 2.1. Study Area and Policy Context

This paper examines the case of London, made up of 33 local authorities or boroughs under the umbrella of London Greater Authority (GLA). London is a city with an extensive network of greenspaces, many climate vulnerabilities and raising health inequalities. Its policy and governance frameworks addressing greenspace, climate and public health are complex and fragmented and pose challenges to integrated approaches. Since 2019, 28 out of 33 London boroughs have declared climate emergencies and see urban greenery as a means to offsetting emissions, especially through initiatives such as tree planting but also living roofs and walls, introducing new greenspaces and planting grasses on road verges [37]. Greenspace is also seen as an opportunity to create ‘cool spaces’ in response to London’s periods of hot weather by providing places for respite and recovery [38]. Moreover, borough-level health and wellbeing strategies aim to make greenspaces accessible to all, aligning with London’s goal for every resident to live within a 10-min walk of a greenspace. These efforts also focus on improving the quality of London’s air, water and biodiversity, highlighting the interconnectedness of greenspace, health and environmental goals.

### 2.2. Islington

**Figure 1:**
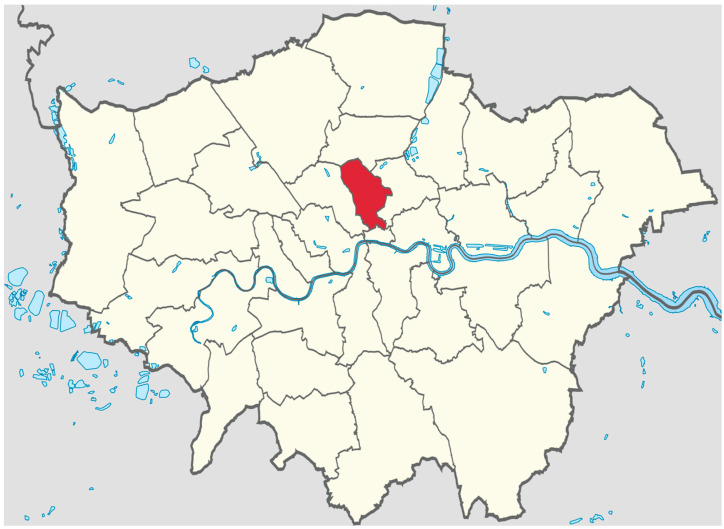
The 33 boroughs of Greater London Authority, with the London Borough of Islington highlighted in red (Source—https://upload.wikimedia.org/wikipedia/commons/4/46/Islington_in_Greater_London.svg, accessed on 21 February 2025).

The scope of the study’s focus and data collection started in the London borough of Islington, Islington thereafter, and then extended to the wider context of London and other London boroughs. Islington is located in North London (Figure 1), 15–20 min by underground from Covent Garden. It is one of London’s smaller boroughs—approximately 15 square kilometres and 220,000 inhabitants—and home to notable socio-economic disparities; while it boasts affluent areas, it also experiences high levels of deprivation. It is a densely populated municipality, with 14,833 residents per square kilometre, which makes it the second-most densely populated local authority in England.

Islington has been selected as a starting point for the research for several reasons. First, the author has worked with Islington before, and a formal partnership exists between their institution and Islington; this offered logistical ease for the start of fieldwork. Second, Islington declared a climate and ecological emergency in 2019, recognising its vulnerability to climate risks such as heatwaves and air pollution, and it is among the six London boroughs most susceptible to climate change. Third, when compared to other London boroughs, Islington has a lower proportion of greenspace per resident, reflecting its dense urban environment, with greenspace only occupying 13% of its total area and 30% of its households having no access to private or communal gardens. Thus, Islington faces significant challenges in expanding its greenspace, and so, it provides a good example for the challenges and opportunities of introducing greenspace in dense urban settings. Fourth, Islington is home to one of four Health Determinants Research Collaborations (HDRCs) in London, set up to boost health research capacity and capability within Islington and prioritise evidence-based interventions to reduce poverty and health inequalities in the borough.

Finally, and most importantly, Islington views greenspace as a core component of its climate change and health inequalities strategies and has developed innovative greenspace policies and adaptation projects, making it suitable for evaluating existing initiatives and their health co-benefits. This includes the Islington Greener Together Programme, which introduces measures—such as increasing tree canopy cover, biodiversity enhancement and urban greening initiatives—to improve air quality and deliver sustainable urban drainage and urban cooling while encouraging active travel, safer streets to schools and more liveable neighbourhoods [39]. Additional examples include the Highways Greening Accelerator Programme, a joint initiative between Islington and a neighbouring borough (Camden) that targets the greening of highways; Green Finance for Islington Pocket Park Framework (PPF), discussed later and Greener Together Champions, which engages residents in greening action for climate change.

### 2.3. Research Approach

The study adopts a policy-engaged research approach, which emphasises addressing policy-related problems or areas of interest while seeking to support live policymaking processes. This approach entails a collaborative and participatory process that actively involves policymakers throughout the research process. By co-producing the research focus and questions with policymakers, transdisciplinary perspectives were integrated throughout the process to ensure that the research remained contextually relevant and directly addressed real-world challenges.

Policy-engaged research is a type of action research and different from policy engagement. Policy engagement is an umbrella term describing the many ways that researchers and policymakers connect and explore common interests at various stages in their respective research and policymaking processes. In contrast, policy-engaged research places a strong emphasis on the collaboration and the co-production of knowledge with policymakers. Unlike policy engagement (i.e., from academia to policy), it prioritises a two-way dialogue with policymakers and integrates their expertise to create actionable outcomes. Such an approach is particularly effective in tackling urban sustainability issues, where the diversity of perspectives is essential to addressing the complex interplay of environmental, social, economic and political challenges. Scholars argue that engaged research strengthens both the relevance and legitimacy of findings, fostering greater uptake by policymakers and practitioners [40]. Additionally, it enhances the capacity of all stakeholders to identify and implement innovative solutions that are socially and environmentally equitable and acceptable [41].

The study built on the author’s previous work on the Green Finance for Islington Pocket Parks Framework (PPF). The PPF received government funding to explore how Islington’s 300+ dead ends and stub roads could be transformed into pocket parks. Within the PPF, the author supported Islington by spatially mapping the intersection of greenspace, climate risks and health exposure; identifying and categorising the climate and health benefits of pocket parks and recommending criteria for prioritising pocket park interventions [1]. This work opened up a window into relevant municipal policy and practice and highlighted a need for better understanding the intersection of climate adaptation, public health and greenspace intervention at the local level. It also resulted in mutual learning and shared ownership of both the process and outcomes, fostering a strong partnership between researchers and Islington, enabling a thorough understanding of the challenges that local policymakers encounter in practice. Over time, this collaboration scoped out three areas of common interest: (1) the practice and alignment of greenspace, climate adaptation and public health policy, discussed in this paper; (2) the use of greenspace to mitigate urban heat islands’ effects on school children and (3) the mental health and wellbeing outcomes of greenspace interventions, explored in subsequent papers elsewhere.

### 2.4. Methods

The paper followed a two-step data collection and used qualitative methods: first, semi-structured interviews with Islington to gain in-depth insights into one London borough’s practice and policy alignment, and second, a focus group with London boroughs and GLA to understand how Islington findings resonate with and are generalisable to the wider context of London.

*Semi-structured interviews* were conducted first with eight municipal officers (coded P1 to P8) in Islington, with roughly two-thirds (2/3, 5 interviewees) representing departments such as greenspace, climate action, highways and transport, spatial planning and environmental services and one-third (1/3, 3 interviewees) public health services. The participants included staff at early, mid or senior career levels and were selected based on their expertise in the researched area. The interviews were conducted in the summer of 2024, lasting between 40 and 90 min each, and were mainly virtual and guided by open-ended questions structured under three broad topics: what is the practice and current greenspace initiatives at the borough level; municipal data, metrics and evaluation frameworks and how greenspace, climate adaptation and public health policy are integrated on the ground. The interviews were audio-recorded and fully transcribed for analysis.

Following the interview data analysis, a *focus group* was organised, which took the form of a half-day (4 h) in-person roundtable with representatives of 18 out of 33 London boroughs (coded RT) of various levels of seniority and representing relevant departments from across local government area of work, with about one-third (1/3, 6 participants) from public health services. The roundtable began with a presentation of the Islington findings, from which only themes relevant across all boroughs were selected and discussed. Discussions were guided by an expert facilitator and audio recorded. A semi-structured discussion protocol was used to guide the facilitation, using PowerPoint presentation prompts to focus discussions around two main topics: how your borough/experience resonates with the Islington findings and what is the potential (challenges and opportunities) for scaling greenspace initiatives that are integrated with climate adaptation and public health targets.

All participants were provided with informed consent forms via email, which were signed before the interviews and roundtable discussions took place. To maintain anonymity, only their assigned codes are used throughout the paper. Given their close association with Islington and London boroughs, disclosing details such as their specific area of work/expertise, seniority level or borough association could lead to identification.

### 2.5. Data Analysis

**Figure 2:**
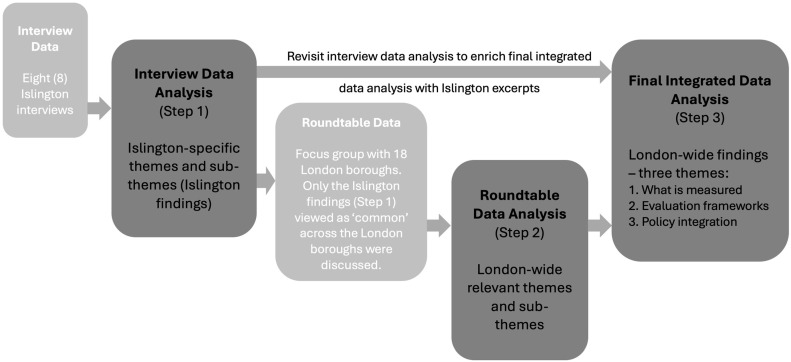
Three-step data analysis: Step 1—interview data analysis; Step 2—roundtable data analysis; Step 3—integrated data analysis.

The data analysis was conducted inductively, allowing the main themes to emerge directly from the data by using thematic analysis [42]. This approach facilitated the identification, categorisation and interpretation of patterns across the data collected in three steps, which are presented in Figure 2.

First, the Islington interview transcripts were manually coded using Microsoft Word for reading through data and word searches and Excel for the recording and organisation of codes, themes and sub-themes, as well as keeping track of relevant excerpts and transcripts. Manual coding was preferred and considered to provide more flexibility and control for the researchers and a deeper understanding of the context and was not too time-consuming due to the relatively small amount of data collected (i.e., eight interviews). The interview data was organised by one researcher according to the three key areas explored during the interviews, and themes emerged by aggregating and summarising the responses. Following this, two researchers had an open discussion on question coding and thematic decisions, reconciled their different perspectives, revisited the data together and reached consensus through iterative review and negotiation. This resulted in interview data analysis, organised by themes and sub-themes (Step 1).

Second, the analysis of the focus group (roundtable) data also employed a manual thematic analysis approach consistent with that used for the interviews. In contrast to the interviews, the roundtable discussions created a more dynamic environment, allowing participants to debate issues, expand on topics and share both collective insights and personal examples from their respective boroughs. This process provided an opportunity to agree on themes and sub-themes developed from the interview data that resonated with or were common across London boroughs and develop/refine those further by incorporating perspectives from participants across various boroughs. The roundtable data analysis was also reviewed and questioned in an open discussion between two researchers, resulting in the final roundtable data analysis (Step 2).

Finally, the final roundtable data analysis was integrated and enriched with excerpts from the interview data analysis and organised under the three broad headlines presented in ‘Results’, i.e., what is measured, evaluation frameworks and policy integration (Step 3). This integrative approach facilitated the contextualisation of the Islington findings within the broader context of London. Only Islington findings that were viewed as common across London boroughs by the roundtable participants are presented and discussed in detail in the following sections. Hence, we are confident that the findings discussed here are relevant to most London boroughs, as well as many other metropolitan contexts in the UK and elsewhere.

### 2.6. Limitations and Implications for Generalisability

This study may be perceived as having at least three limitations. First, the initial interviews were conducted in one London borough, Islington. While this provided valuable insights, it could be argued that the findings are less generalisable to other boroughs with differing geographic, environmental, socio-economic or political contexts. However, this initial focus was intentional and aligned with the policy-engaged approach adopted in the study. More specifically, the author sought to immerse themselves in the unique context of Islington, starting with a broad research focus to identify real-world problems and develop specific, contextually relevant research questions of mutual interest to both the researchers and the borough. Subsequently, both the researcher and Islington questioned whether similar issues existed in other boroughs and were interested in collective opportunities and challenges for addressing them. To investigate these questions, the study incorporated a focus group designed to test and critique the ‘Islington problems’ within a broader municipal context. This approach provided a platform for dialogue, enabling participants to explore the relevance of these issues to the wider framework of municipal practices in London.

Second, the eight interviews conducted may be perceived as a relatively small dataset. While the selection process prioritised participants with relevant expertise, it may have excluded perspectives from less-involved departments or external stakeholders (e.g., private funders and community representatives), the latter, however, not being the focus of the research. The relatively small number of interviews is grounded in the organisational structure of the borough, ensuring representation from all relevant divisions. Local governments in England typically consist of up to 10 principal departments, and interviewing 8 representatives per borough aligns well with this structure. Furthermore, studies focusing on specific local authorities often conduct between 5 and 15 interviews per authority, usually targeting a broader range of stakeholders (e.g., policymakers, council members, administrative staff, etc.) than this study. The narrowly focused research topic, targeted questions and knowledgeable nature of the interviewees have ensured sample saturation and that the research findings that emerged did not present significant gaps. Similarly, the focus group included participants from 18 out of 33 London boroughs, which may be seen as not fully representative of all views and experiences across the city. While this may not capture every perspective, the sample size is sufficient to achieve saturation due to the specific expertise of the participants. The majority were mid- to senior-level policymakers with extensive experience across multiple local authority settings. This professional breadth enabled them to contribute well-informed, comparative insights drawn from diverse municipal contexts, ensuring a wide-ranging discussion.

Third, focus group findings have several limitations, with implications for findings representativeness and generalisation. Dominant voices in the focus group may monopolise the discussion. To avoid this, an experienced moderator was used, and the roundtable discussion was guided by questions with round robin and 2 min responses to give all participants a chance to speak in turn. After the session, the researchers followed up with separate emails to encourage further input from individuals, and two additional responses were received. The seniority of participants can be another limitation and introduce bias, as perspectives from more junior municipal staff might offer different insights. However, the seniority of the participants in the focus group was intentional to capture strategic (across London) and decision-making perspectives; it is acknowledged, however, that these may not fully reflect municipal challenges, i.e., an emphasis on high-level strategies over practical issues or positive representation of the policies they helped to design. Variation in participants’ context (i.e., municipal priorities, greenspace availability, etc.) can frame the participants’ experiences and responses. To recognise this and ensure findings that were transferable, the research design (e.g., focus group) enabled them to commonly agree on themes for discussion that ‘spoke to’ all participants and were viewed as cutting across municipal differences.

To sum up, the research approach was intentionally designed to be immersed in Islington’s unique challenges, allowing for the development of contextually relevant research questions and findings. Such findings were then tested in the broader municipal context of London through a focus group discussion to identify themes that resonated across diverse municipal contexts and decision-makers. Hence, this approach enables the applicability of key findings across London boroughs. While rooted in the London context, these findings can also be transferred to other cities in the UK and abroad. The focus on senior policymakers enables insights into decision-making processes that are often shared among cities. While limitations in fully capturing local or grassroots perspectives are acknowledged, the paper’s emphasis on shared municipal opportunities and challenges in large cities frames an approach that informs the multifunctionality of greenspace, climate change and public health interventions in urban areas beyond London, including international contexts facing similar sociopolitical and environmental complexities. However, limitations remain in fully applying these insights to non-European urban contexts, particularly in the Global South, which faces challenges such as rapid urbanisation, limited financial resources and weaker municipal capacity. Greenspace adaptation efforts in these settings often rely on community-led projects, nature-based solutions tailored to local needs and multifunctional spaces that address both climate and social vulnerabilities. In addition, while European cities advocate systematic urban greening integrated into spatial planning, non-European cities often adopt more adaptive, decentralised approaches to maximise health and climate benefits with available resources.

## 3. Results

The following results were developed under three themes (what is measured, evaluation frameworks and policy integration) that emerged from the three-step data analysis process described in Figure 2 and frame the research question and aims.

### 3.1. What Is Measured

Three broad headlines summarise the findings in this section, briefly outlined in Table 1:

- current indicators that look at municipal greenspace-related indicators currently in use, mainly describing physical and environmental characteristics of greenspace (e.g., canopy cover and number of trees) but also activities related to greenspace (e.g., community volunteering and green champions).
- desirable indicators that note what other indicators measuring greenspace’s multifunctionality should be measured (e.g., carbon/heat/flooding mitigation, biodiversity enhancement and social) and emphasise the importance of socially relevant greenspace indicators in demonstrating social, political and investment values. Interestingly, health indicators were mentioned but no examples given.
- measurement challenges that identify municipal barriers to measuring the multiple benefits of greenspace, including lack of data or access to data, lack of skills and capacity and also methodological and technical bottlenecks.

### 3.2. Current Indicators

Most participants agreed that the current municipal greenspace metrics focus on physical and environmental characteristics of greenspace such as canopy cover, the creation of new greenspaces and tree planting. Interviewee P4 noted:

*...with trees, we’re looking at things like canopy cover.... The only data that we’re collecting now is the amount of new greenspace delivered, the ‘per squared meter’…so, in terms of squared meters of highways and land that’s turned into greenspace that’s what we’re calculating and keeping track of, and recording.*

Interviewee P2 explained that the existing metrics also monitor ‘activity’ such as the number of volunteer hours or green champions, hence focusing on output-based data. However, participants felt that the current emphasis on physical and environmental measures, while valuable, fails to capture broader greenspace benefits such as carbon sequestration or heat mitigation and health co-benefits, including physical and mental health and wellbeing outcomes. Interviewee P5 noted:

*I would welcome advice on... better metrics that are more specifically highlighting health benefits or carbon benefits.*

### 3.3. Desirable Indicators

Participants discussed the need for additional indicators that are potentially more valuable for borough-level decision-making, including tracking activities like pollination or urban drainage (P1). Interviewee P2 highlighted the potential for using modelling to measure heat and pollution mitigation, which could offer further insights into the broader environmental benefits of greenspaces, while interviewee P6 noted:

*...there’s plenty more work to be done... around the health benefits of greenspace...*

*and that despite the significant groundwork and readily available reports and evidence available, there is ample opportunity to build on these efforts to make further progress.*

Besides validating the views on greenspace measurement at the municipal level, RT participants emphasised the importance of incorporating social indicators into the assessment of greenspace initiatives, recognising their role in contributing to equitable and sustainable urban development. RT participants noted that social value measures have the potential to inform decision-making, and such measures collected before and after interventions could validate investments and increase social acceptability, and leveraging greenspace projects to engage young people could lead to nature-based careers and fostering biodiversity stewardship. Another RT participant highlighted the need to link the physical, environmental and social benefits of greenspace, because demonstrating ‘social value’ was viewed as being highly valuable for attracting private green finance and ensuring investments that align with environmental, social and governance (ESG) goals.

### 3.4. Measurement Challenges

Participants noted several challenges in capturing the multiple benefits of greenspace and measuring broader climate and health outcomes. A main challenge was the lack of municipal data, or rather, access to municipal data, and so the reliance on anecdotal evidence instead of robust, empirical data, thereby undermining evidence-based decision-making. The twofold challenge of municipal capacity and skills—where lack of financial and human resources (including high staff turnover) limits the borough’s capacity to collect and maintain local data/datasets—and a municipal focus on delivery and fast-paced-short-term execution timeframes—which are prioritised at the cost of establishing solid and comprehensive data collection frameworks—was also mentioned.

*It is so complex to put in a greenspace, dealing with objections, getting public support, getting all of the engineering measures together...doing all of that, that is such a challenge. Then to be there and start monitoring the effects of it afterwards. That’s just like something that is sometimes seen, in short-term thinking, as an opportunity cost to being able to just get on and do some more greenspaces.*

*(RT discussions)*

Methodological challenges, such as ‘what data’ and ‘how data are collected’ to align with/feed into the measurement of climate and health benefits of greenspace adaptation, were finally noted. Moreover, RT participants noted that most boroughs lack the skills and capacity to draw on existing datasets about greenspace, climate risks and health that may be available in London.

### 3.5. Evaluation Frameworks

The findings on greenspace evaluation frameworks are grouped under two sub-themes, summarised by Table 2:

- existing frameworks note the limited and KPI-driven nature of the current municipal greenspace measurement and fail to draw on health data due to use constraints but also poor understanding of the health co/benefits of greenspace at the municipal level
- integrated frameworks need integration across the greenspace, climate and health metrics and, ideally, alignment with KPI, SDG and ESG targets

### 3.6. Existing Frameworks

Participants noted that existing evaluations of greenspace were limited, often relying on readily available data and focusing on baseline key performance indicators (KPIs), with municipal practices yet to explore a practical and municipally aligned framework to measure the broader benefits of greenspace. Interviewee P4 noted:

*We always talk about the fact that there are multiple benefits to greenspace, but then we cannot quantify them or maybe we can quantify them, but it’s really complicated and we [the Borough] don’t have capacity or skills to do it.*

It was reported that KPIs were frequently used to monitor greenspace within various municipal teams, although some teams were ‘very KPI-driven’ (e.g., Landscape and Parks Team), while others only used them sparingly (e.g., Greening the Highways Team). Greenspace KPIs were viewed as lacking in depth and being focused on a limited number of well-tested indicators, such as the number of new planted trees and area of new greenspace. One RT participant noted:

*It’s sort of saying that...we’ll deliver 30 greening projects next year when we already know that we’re probably going to deliver 30 greening projects in the next year. So, it’s not very rigorous. A lot of the KPIs we’re using are based on the work that we already are doing.*

Moreover, the current environmental KPIs at the borough level were viewed as tightly tied to carbon emissions reductions and reporting government standards and so not linked to the climate-related benefits of greenspace adaptation. One RT participant noted:

*We currently have corporate KPIs for environment and climate change...we have KPIs on our overall carbon emissions reduction, which is measured by government...but it’s not calculated for greenspace interventions which are mainly associated with climate adaptation and not carbon reductions.*

The task of evaluating the health co-benefits of greenspace adaptation was viewed as even more complex. This was due to concerns about data sharing and privacy, restrictions on how the data can be used and the challenge of directly linking greenspace intervention to specific health outcomes.

### 3.7. Integrated Frameworks

Alternative evaluating or measuring approaches were mentioned, including geospatial mapping, supported by organisations like the European Space Agency. Aligning data collection with global targets indicators, such as those tied to the United Nations Sustainable Development Goals (SDGs) Framework, was also viewed as having the potential to create a more integrated and comparable evaluation framework across greenspace intervention at the municipal level. In fact, the integration of SDGs; key performance indicators (KPIs) and environmental, social and governance (ESG) indicators into municipal reporting processes and performance evaluation was identified as both promising and challenging.

One interviewee noted that KPIs are often ‘set by departments on key services’ and focused on corporate goals (P5), suggesting that SDG indicators and, more recently, ESG indicators were considered separately and not integrated into a department’s target-setting frameworks. The inclusion of ESG-related measures in existing evaluating frameworks was viewed as fraught with tension in the context of balancing private sector profit-driven motivations with public interests, which frame such potential integration. Interviewee P1 noted:

*The ESGs are important but... it’s always difficult... when you’re working with the private sector... how do we square the circle between the two things?...*

Frameworks such as the UK Woodlands Carbon Code were suggested as a model for creating a standardised and accessible framework that will be embedded within the UK policy context and enable businesses and private organisations to fulfil environmental obligations while bridging the gap between regulatory requirements and private sector-driven investment and fostering more effective private–public partnerships.

### 3.8. Policy Integration

The findings on municipal policy integration across the greenspace, climate and health sectors are organised under three headlines and summarised in Table 3:

- Current practice finds that policy fragmentation and silo thinking persist, despite proactive efforts to join agendas such as stewardship charters and demonstrator projects.
- Municipal collaboration, between municipal departments but also municipalities, is noted as a main driver of policy integration, with ‘good practice’ examples involving greenspace, transport, education and public health municipal teams.
- Barriers to integration note competing municipal priorities, such as immediate needs versus long-term goals; net zero focus; public acceptability and financial, time and staff/capacity constraints.

### 3.9. Current Practice

The level of integration between greenspace, climate and health policy and initiatives at the municipal level in London varies across boroughs, characterised by mixed approaches and determined by levels of policy engagement and knowledge among municipal officers. While departments like environment services, transport and public health proactively integrate climate considerations, this often depends on officers’ knowledge and willingness to do so, suggesting uneven practice and siloed operations at the municipal level. Interviewee P2 remarked:

*We all do work in silos, and it’s very easy for that to happen. Trying to get people to understand the impacts of climate change on their users and residents, as well as their health, is not easy nor necessarily well understood.*

Some proactive efforts to integrate greenspace, climate and health policies across municipal departments were also noted:

*We hold a number of different meetings with officers in the Borough to understand how climate change and public health were affecting their work...what sort of challenges were coming up...what would they be able to do about it within their existing kind of resources, budgets and staff... what they’d like to do about it but perhaps needed a bit more help with...*

*(Interviewee P1)*

The RT discussions noted that such efforts can be mobilised via the development of stewardship charters, which could engage young people in the long-term management of greenspace and provide the skills and capacity building in this area. Engaging the youth was viewed to foster leadership, provide pathways for meaningful involvement in greenspace decision-making and address a significant gap in how young users shape and influence urban greenspace. Demonstrator projects were also viewed as another example of joining various interests and areas of municipal responsibility by linking greenspace and public health data to showcase synergies building on the previous success of programmes like Parks for Health in the London boroughs of Islington and Camden.

### 3.10. Municipal Collaboration

Collaboration emerged as the most important determinant of successful policy integration, with participants consistently emphasising its role in breaking down silos between municipal teams. Interviewee P5 noted that the partnership between Islington’s transport and public health teams in the Active Together strategy speaks to how joint efforts can align climate action with public health objectives by ensuring that greenspace is provided with cycle and walking paths and/or well equipped with play or outdoor gym equipment to encourage residents, and especially those likely to be less active, to move more.

*There is also a number of collaborations between the greenspace and natural environment teams and the highways team, which aim to deliver greener and healthier streets through the Active Together strategy...and the early years education team… like Bright Start where we work with schools to improve and encourage kids access to greenspace…discussions have also started with the public health team trying to understand how to use greenspace to tackle health inequalities in the Borough.*

*(Interviewee P5)*

Islington’s Bright Start Programme strengthened collaboration with schools, and between the greenspace and children’s services teams, by using parks for nature-based education and family engagement in outdoor activities through integrated hubs across the borough. Another example, the Parks for Health Programme, a joint initiative between Islington and Camden councils, reframed the role of parks by viewing greenspace as a means to reducing health inequalities in London.

*We’ve tried to align initiatives across teams by showing how interconnected health and climate policies are, and this approach has begun to resonate with departments...we also try to get different teams together to familiarise with specific and more technical knowledge...*

*(Interviewee P7)*

Participants agreed that creating collaborative opportunities to integrate different policy priorities was an important step towards the scalability of local interventions. They also noted that an integrated and strategic policy framework that aligns greenspace, climate adaptation and public health was also necessary to guide effective delivery on the ground:

*The main challenge is that the policy framework isn’t there right now...developing a London-wide policy framework with clear instructions for boroughs who want to engage in this conversation would be extremely useful.*

### 3.11. Barriers to Integration

Two main challenges to achieving policy integration were mentioned by discussants: competing municipal priorities and resource constraints. First, competing priorities among municipal departments still prevail. Boroughs often face the difficult task of balancing immediate social concerns, such as homelessness and provision of affordable housing, with long-term climate or public health goals. As Interviewee P5 put it, the urgency of providing housing development conflicts with its carbon-intensive nature:

*Building a new home is a carbon-intensive thing and it will generate more carbon emissions...but, if it is also important for people’s overall health and wellbeing.*

Interviewee P7 reflected on the tension between competing goals and the scarcity of urban land:

*When you’ve got competing policy goals, which one takes precedence? There’s competition for urban land in general, which brings our policy ambitions into conflict most of the times.*

Such competing priorities were also viewed in RT discussions as being further complicated by public and social acceptability concerns over issues such as loss of parking, littering and loitering associated with greenspace use.

Second, resource constraints such as financial or budgetary limitations, staff shortages and time pressures were viewed as major impediments to the integration of greenspace, climate and health interventions at the municipal level, as interviewee P3 put it:

*A lot of departments in the Council are very stretched because they must deliver certain services, often on limited budgets or within poorly resourced teams… Adding in other considerations, around climate, adaptation or health is always going to be a challenge.*

The more recent UK emphasis on net zero targets was also seen as an additional pressure because of its focus on climate mitigation and carbon emission reductions at the expense of climate adaptation measures, as noted by interviewee P2:

*Resources have been focused on Net Zero goals, and now adding in adaptation work is an ongoing challenge.*

Participants noted that funding for the net zero project pipeline exists for all councils, with a focus often on large-scale projects. However, greenspace projects are not funded through clear channels and require alternative delivery mechanisms like special purpose vehicles (SPVs) or partnerships with organisations such as the National Trust, UK’s conservation charity that protects historic places and greenspace. Driving such efforts at the borough level was viewed as challenging, and a number of options were suggested, including a GLA-governed partnership to unlock funding for projects that address both greenspace delivery and biodiversity goals and cross-council partnering with housing and transport teams to align greenspace with housing development and transport projects, potentially supporting both net zero and infrastructure delivery goals, as well as dispelling some of the funding constraints and investor hesitance associated with greenspace projects.

## 4. Discussion

The London example confirms that, despite synergetic relations, urban policy and practice around greenspace, climate adaptation and public health are fragmented and disconnected at the municipal level. This is the result of very different approaches to policymaking and practice in these three urban sectors. Traditionally, health and climate interventions are driven by evidence-based policymaking and evaluations to identify the ‘best solutions’, which rely on hard data and measurable outcomes. However, such solutions have been criticised for often overlooking the complexity of urban policy and decision-making processes [43,44]. In contrast, greenspace interventions are better framed by the complexities of urban processes but have been evaluated less for their climate and health co-/benefits. The evidence base for policymaking in this area remains limited and incomplete, mainly relying on soft data and propositional outcomes [45]. Integrating greenspace, climate adaptation and health policies and practice in cities to achieve greenspace adaptation with health co-benefits requires harmonising these two approaches.

This is important for informing policy frameworks for greenspace adaptation with health co-benefits globally and has two implications for urban settings elsewhere. First, developing comprehensive urban data and monitoring frameworks for the multifunctionality of greenspace that are integrated with climate and health data and frameworks is important for the implementation, financing and, ultimately, scalability of urban greenspace adaptation with health co-benefits in our cities. Such development aligns with the current debates on the data-driven governance and the multifunctionality of urban greenspace. Second, such integrated and data-driven greenspace–climate–health governance frameworks can support city planning to prioritise urban interventions by targeting the ‘most vulnerable’ areas first but also attract investment by providing a clear indication of measurable outcomes and a pipeline of ‘investment opportunities.’ These are discussed in detail in the following section.

### 4.1. Data-Driven Governance for Greenspace Multifunctionality

The implementation of greenspace multifunctionality remains fragmented and slow, and greenspace is underutilised as a strategic and multi-benefit tool by local governments. Greenspace intervention is often viewed as a high-cost option at the municipal level, where immediate financial concerns overshadow its long-term multifunctional value. While the climate and health benefits of greenspace are generally acknowledged at the municipal level, the lack of—or, rather, access to—robust data and indicators to monitor and measure these outcomes undermines their value. This aligns with previous studies that have identified a disconnect between local climate action priorities and the contributions of nature-based solutions [28] but also municipal silos and little collaboration between municipal departments [24,31]. Moreover, local governments’ emphasis on carbon reduction and net zero mitigation targets, though critical, often operates independently from the narratives of nature-based cities and adaptation priorities [46] and lacks an understanding of health co-benefits [8].

An overreliance on the physical and/or environmental indicators of greenspace seems to persist at the municipal level, with insufficient attention to climate, health and other measures. This focus is also observed in the existing literature, which mainly measures the biophysical dimensions of greenspace [26]. Three broad categories of greenspace metrics are widely employed by current studies, including the large-scale monitoring of greenspace, such as the Normalized Difference Vegetation Index (NDVI), which uses satellite data or drone imaginary to assess the overall urban greenness or green cover, allowing for easy comparisons between cities and urban areas; accessibility of greenspace, gauged by indicators such as ‘access to greenspace’ and ‘proximity to greenspace’, i.e., greenspace within 400 m or a 5 min walk as required by SDG 11, Target 11.7 and London Plan’s 800 m or 10 min walk to a greenspace; and tree metrics, including indicators such as tree coverage, tree density and tree type.

Many cities have adopted general sustainability, climate or health metrics and indicators; however, integrated, spatially and greenspace-relevant measures remain underdeveloped. For example, the literature provides many specific examples of health metrics that could be incorporated into greenspace evaluations, including metrics associated with levels of physical activity; reductions in associated heat- and air pollution-related illnesses; improvements in mental health and wellbeing outcomes (e.g., stress, anxiety, social integration and cognitive development) and hospital admissions [8]. Integrating greenspace projects with measurable health goals could strengthen their strategic role within urban resilience frameworks, also promoting transparency, joint understanding and local ownership [47]. However, most health datasets often lack in or lack access to spatial resolution (i.e., street and postcode level) to describe complex microclimates in cities, and differences in measurement techniques can hinder comparability and integration across urban areas. In addition, privacy regulations can restrict the granularity of data sharing, and socio-economic, demographic and geographic conditions can further confound outcomes—for example, health benefits observed at the local level do not always scale to citywide outcomes because of socio-economic, but also geographic, variations [48]. Such challenges complicate the design, evaluation and optimisation of greenspace interventions aimed at climate adaptation while improving public health.

There is agreement across London’s municipalities that a robust database to inform decision-making across greenspace–climate–health interventions is still missing and that climate and health measures of urban greenspace intervention need to be included in the existing urban data platforms [49]. This aligns with the current global calls for data-driven urban governance [50] but also exposes critical contradictions. While cities around the world are investing in data infrastructure to inform evidence-based decision-making, London included, the effective utilisation and quality of such data infrastructure may remain inconsistent, siloed and not fit for purpose.

Addressing such contradictions may require the integration of data-driven governance models with participatory governance models and citizen science. Such integration could promote transparency, shared understanding and, in the broader sense, community ownership. Examples include Amsterdam’s smart parks with real-time data and community feedback where AI-powered dashboards analyse real-time environmental and user data and adjust such data based on citizen inputs to ensure urban greenspaces match community health and ecological needs [51]; Copenhagen’s crowdsourced active mobility initiative where citizens contribute cycling route data via mobile tracking apps and the city uses AI to improve bike lane design and integration with greenspace [52] and China’s sponge cities program, which promotes community-led flood-resilient greenspace initiatives by using citizen-reported flooding data, satellite imaginary and AI design [53].

Participatory data collection and involving communities in the design and evaluation of greenspaces can also address the identified problem of a lack of granular data at the municipal level but also earlier criticism about the exclusion of marginalised voices in greenspace governance processes [54]. Citizen science initiatives, where residents monitor the use and quality of greenspaces but also their mental health and wellbeing, for example, could empower communities while generating granular data to add to the existing urban data platforms and inform municipal governance frameworks. For instance, local governments might use smartphone applications and digital twins to track and model, respectively, greenspace usage patterns alongside climate risks and health exposure, helping to identify ‘problematic’ areas and inform or prioritise intervention efforts.

The shift toward data-driven governance also opens up new funding opportunities for greenspace projects, as robust data collection can feed into evaluation metrics, which, in turn, can demonstrate a return on investment in alignment with ESG strategies, which have emerged more recently as an approach to framing sustainability commitments in the private sector. For example, the UK Municipal Bonds Agency’s (MBA’s) Sustainable Finance Framework enables local councils to issue ESG bonds aligned with the Green Bond Principles to attract private investments in projects of public interest [55]. Such bonds could be issued for greenspace projects with one caveat: public–private partnerships (PPPs) have previously faced criticism for their handling of public and private interests. To address these concerns, local governments should establish transparent criteria for project selection, engage communities in decision-making processes and implement rigorous monitoring mechanisms that balance diverse interests effectively.

This study did not look at the economic and financial implications of undertaking urban greenspace adaptation with health co-benefits; however, this ‘theme’ surfaced again and again throughout our discussions on London boroughs. References were made to the monetary value/cost of taking/not taking climate action and healthcare costs and savings of greenspace, with associated benefits or damage estimates for insurance companies, urban utilities or the National Health System (NHS). This is, for sure, an area of both interest and concern to other urban contexts and should be further unpacked in future research.

Economic and financial considerations are integral to urban governance frameworks, shaping policy priorities, greenspace investment and delivery. Urban decision-makers only require an order of magnitude estimate of added value and/or damage costs to inform policy and interventions on the ground [9]. Urban data platforms should be able to assign such estimates, as it is often impractical or too costly for local governments to create or draw on the in-house capacity in such technical areas [56]. Our participants suggested building partnerships with other municipalities and working closely with local knowledge-based institutes and universities that can provide the necessary technical expertise. The results suggest that establishing common frameworks for monitoring and financing the integration of greenspace, climate adaptation and public health initiatives are major issues that need to be addressed at the municipal, city and regional levels. This would require policy integration and reforming the current governance landscape away from siloed and path-dependency municipal practices to multi-actor, multi-level and integrated data-driven governance frameworks.

### 4.2. Policy Integration and Spatial Planning

Despite many cities adopting sustainability, climate or health policy and/or monitoring frameworks, an integrated greenspace–climate–health policy framework for ‘city action’, supported by indicators that capture multiple benefits, is currently lacking in London and, to the best of our knowledge, elsewhere. Integration can be achieved by leveraging advanced methodologies, such as remote sensing technologies, geospatial analysis and epidemiological survey data, and datasets can be combined through machine learning and GIS-based spatial analysis. Such a framework could guide urban decision-making processes and prioritise intervention and target investment but also enhance urban planning strategies, ensuring that greenspaces are equitably distributed and optimally designed to support environmental sustainability and public health. More importantly, it should draw on an interdisciplinary approach, combining environmental science, urban planning, public health and social policy perspectives, among others [24].

**Figure 3:**
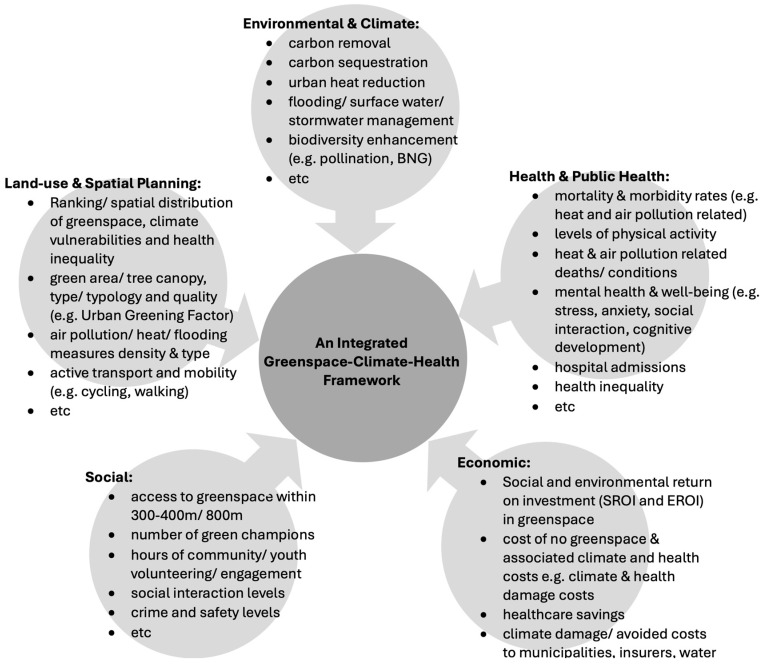
An integrated greenspace–climate–health policy and measurement framework, with multiple greenspace co-/benefits and metrics/indicators to consider.

A hypothetical integrated urban policy framework for greenspace adaptation with health co-benefits is presented in Figure 3, including references to the multiple dimensions and benefits of greenspace. Its variables or indicators should be aligned with KPI targets—such as tree canopy cover, reduction in air pollutants, greenspace use, reduced hospital admissions, etc.—but also existing SDG and/or ESG targets for a better alignment with broader urban sustainability goals. For example, linking air quality improvement (SDG 11—Sustainable cities and communities, Target 11.6—PM levels) with tree canopy expansion (SDG15—Life on land, Target 11.1—forest/tree area as proportion of land area) and pollution reduction targets (SDG 3—Good health and wellbeing, Target 3.9—deaths attributed to air pollution relevant conditions) support spatial, environmental, climate and public health policies.

As suggested by London municipalities, such integrated frameworks could be governed at the city or region level by multi-stakeholder multi-level task forces led by the city—such as the Greater London Authority (GLA)—or regional government and instrumentalised to foster cross-municipal partnerships between, for example, housing and transport teams to align greenspace with housing development and transport projects and potentially to support both net zero and infrastructure delivery goals, as well as dispelling some of the funding constraints and investor hesitance associated with greenspace projects.

Political leadership and will are the main challenges of any governance model. While the integrated framework provides strategic oversight and efficiency, its success ultimately depends on the commitment of elected leaders to sustain long-term collaboration and integration across different municipal departments and levels of government. Another challenge is the election-driven decision-making that shapes a municipal government, whereby elected politicians may favour short-term, high-impact projects over long-term frameworks that require sustained investment and complex coordination. Greenspace projects, despite their well-documented climate and health benefits, often do not generate immediate electoral gains. If a newly elected administration prioritises different urban policies, there is a risk that the framework could be abandoned or significantly altered before yielding any tangible results. This uncertainty may also discourage private investors from supporting greenspace projects, as they may perceive the political landscape as too unstable for long-term commitments. Nevertheless, significant political benefits remain, including enabling policymakers to demonstrate effective governance and position themselves as leaders in sustainable urban development and responsive, solution-driven politics, and politicians who champion such initiatives can leverage visible improvements—such as enhanced public health, increased climate resilience and better-connected communities—as tangible achievements that resonate with voters, reinforcing their political credibility.

While the political dimensions of the integrated greenspace–climate–health policies and frameworks shape their feasibility and long-term viability, their implementation in urban areas carries important spatial implications. Effective implementation requires an integrated approach to land use planning, ensuring that greenspaces are strategically embedded within the urban infrastructure rather than treated as peripheral or isolated elements. This necessitates a shift toward spatial policies that prioritise the multifunctionality of greenspaces, serving not only as aesthetic and recreational areas but also as climate adaptation tools and public health assets.

Urban planning emerges as a critical yet underutilised tool for fostering policy integration for greenspace multifunctionality. Indeed, it has been viewed as a structured and strategic platform to address fragmented or uneven spatial outcomes caused by siloed municipal practices, mitigate land use tensions arising from competing urban priorities and facilitate mechanisms for scalability [57]. It is also an effective tool for addressing future challenges, such as those related to climate and public health [58], and has engaged in collaborative practices before [59].

The concept of multifunctionality is well established in urban planning, particularly in relation to green infrastructure and ecosystem services [60]. It is also applied to land use planning to advocate for a shift from monofunctional land uses to multifunctional practices that support diverse uses simultaneously [61]. Hansen et al. (2019) identified two key scales for delivering greenspace multifunctionality in urban planning: the design and location of greenspaces at the development scale and the integration of greenspaces as a planning principle at the broader city or regional level [62]. This is particularly important in cities where greenspace is scarce and competition for land use is high. By embedding climate and health objectives into spatial policies, planners can intentionally support net zero targets through strategies such as carbon removal and sequestration while also enhancing physical access to greenspaces. This not only reduces health inequalities but also improves mental health outcomes and promotes active mobility.

Urban planning is well positioned to manage conflicts and to address disparities by providing a strategic, place-based framework that integrates diverse urban policy goals while prioritising equitable outcomes. With its capacity to coordinate land use, infrastructure and resource allocation, spatial planning can mediate competing priorities at the municipal and city levels, such as balancing housing provision with greenspace investment, urban land scarcity and resisting development pressures to preserve greenspace for climate resilience and health benefits. For example, spatial planning policy can impose greenspace as a requirement of any housing development, facilitated by tools such as the Urban Green Factor [63]. Moreover, by identifying urban areas and communities of the greatest need, spatial planning can target interventions that enhance access to greenspace, and by working across municipal departments, it can nurture cross-departmental collaboration, which, in turn, leads to breaking down siloes and promoting common agendas that integrate climate adaptation strategies with public health goals and greenspace delivery.

Spatial planning can also identify trade-offs and conflicts that may arise between different policy objectives or components of the urban system, which is crucial for minimising the unintended consequences of greenspace solutions that have gained traction in recent years [8,64]. DePietri (2022) categorised such trade-offs into four categories: ecological, such as choosing between greening initiatives and water management or managing stormwater while maintaining landscape connectivity; socio-economic, including impacts on health, safety, wildlife interactions and the risk of green gentrification; technological, such as tensions between green and grey infrastructure and the associated maintenance costs; and institutional, involving competing priorities such as housing development in the short term versus GHG reductions in the long term [65]. For example, planning tools such as the Urban Green Factor can balance out such competing priorities by requiring housing developments to include multifunctional greenspace [63]. Moreover, by examining factors like urban land scarcity, social acceptability and environmental impacts, it can also reveal conflicts, for example, balancing greenspace preservation with development pressures or mitigating the carbon footprint of construction activities.

## 5. Conclusions

Three key findings have emerged from this London study. The articulation and measurement of greenspace benefits at the municipal level remain limited, with emphasis placed on a narrow set of indicators, such as canopy cover and the number of new trees or greenspaces. The current evaluation practices are shaped by local and national KPIs but fail to capture greenspace multifunctionality or align with climate- and health-related outcomes. Municipal policies and practices for climate adaptation with health co-benefits are fragmented and insufficiently integrated; while collaboration fosters progress, competing municipal priorities and resource constraints pose significant challenges.

These findings have important implications for policy and practice beyond the London context. The lack of integration between the greenspace, climate adaptation and public health sectors is driven not only by municipal siloing but also by historically different policymaking approaches in these fields. Addressing these challenges requires integrated policy and evaluation frameworks that facilitate the implementation, investment in and expansion of urban greenspace adaptation projects with health co-benefits. Such frameworks should be grounded in city- or regional-level data-driven models of governance that are complemented by citizen science to enhance representation, transparency and community ownership. Furthermore, they can be operationalised through spatial planning strategies aligned with broader sustainability objectives, ensuring greenspaces are equitably distributed and optimally designed to support climate resilience and public health.

Several key areas for future research are highlighted at the end of this paper. There is an urgent need for integrated urban evaluation frameworks that assess the climate, health and broader benefits of greenspaces. While this paper proposes one example, further research should explore additional models and examine their alignment—or lack thereof—with existing KPI, SDG and ESG frameworks. In addition, research should also look at data-sharing and privacy challenges that hinder the inclusion of health data into greenspace evaluations; document innovative governance models that integrate data-driven and participatory approaches and, deepen the understanding of greenspace’s role in mental health and wellbeing as well as the management of chronic conditions such as diabetes, cancer and cardiovascular and respiratory diseases. Finally, investigating the economic and financial implications of such projects is essential for informing policy and investment decisions.

## Figures and Tables

**Table 1:** What is measured: current indicators, desirable indicators and measurement challenges.

What Is Measured	Findings
Current indicators	The main focus is on a limited number of physical and environmental indicators, e.g., canopy cover, area of new greenspace and number of planted trees
	Some other relevant indicators include number of greenspace community volunteering hours and number of green champions
Desirable indicators	What else should be measured: Broader benefits, e.g., greenspace’s role in carbon sequestration, heat mitigation, urban drainage management, biodiversity restauration (i.e., pollination) Health co-benefits were mentioned but not substantiated Social benefits
	Social indicators should be measured: Demonstrate ‘social value’ and incentivise green investment and alignment with ESG strategies in the business sector Support municipal decision-making Increase social acceptability Encourage youths to engage in nature-related skills/training and stewardship
Measurement challenges	Lack of municipal data and/or access to data
	Lack of in-house capacity and skills to process/work with data
	Methodological, e.g., ‘what’ data and ‘how’ data are/should be collected and ‘by whom’

**Table 2:** Evaluation frameworks: existing frameworks and their limitations and potential integrated frameworks.

Evaluation Frameworks	Findings
Existing frameworks	Limited, with monitoring based on a few measures, e.g., number of new trees, new greenspace area, etc.
	Currently driven by municipal and national KPIs.
	Health data are difficult to incorporate due to data use restrictions and lack of understanding of health benefits/co-benefits of greenspace.
Potential integrated frameworks	No integrated frameworks measuring the multiple benefits of greenspace exist in London.
	Frameworks that integrate KPI, SDG and ESG indicators are promising but challenging.

**Table 3:** Policy integration: current practice, collaboration and barriers to greenspace–climate–health policy alignment.

Policy Integration	Findings
Current Practice	Dominated by ‘silo’ thinking and various degrees of policy alignment
	Joining efforts across municipal silos can be achieved via stewardship charters and demonstrator projects
Municipal collaboration	A main driver of integration; both intra- and intermunicipal collaboration are important
	Good practice exists, with collaboration between greenspace teams and transport/highways, early years education and public health services
Barriers to integration	Competing municipal priorities, e.g., balancing immediate needs (i.e., housing shortage and homelessness) with long-term goals (climate); predominant focus on net zero; land use pressures; public acceptance
	Resource constraints, e.g., financial/budgetary limitations, staff shortages and lack of skills and time pressures, i.e., short-term timeframes

## Data Availability

The raw data (interviews and roundtable transcripts) supporting the argument of this article is not readily available due to privacy and the data being part of an ongoing study. Further inquiries can be directed to the corresponding author.
